# Genome-wide DNA methylation dynamics in carbon tetrachloride-induced mice liver fibrosis

**DOI:** 10.22038/IJBMS.2022.66256.14555

**Published:** 2023-01

**Authors:** Deming Li, Xiaoshu Guo, Wenyu Zhao, Jingyu Jingyu, Cong Xia, Guoying Yu

**Affiliations:** 1 State Key Laboratory Cell Differentiation and Regulation, Henan International Joint Laboratory of Pulmonary Fibrosis, Henan Center for Outstanding Overseas Scientists of Pulmonary Fibrosis, Overseas Expertise Introduction Center for Discipline Innovation of Pulmonary Fibrosis (111 Project), College of Life Science, Henan Normal University, Xinxiang, Henan, China; 2 Department of Physiology, Changzhi Medical College, Shanxi, China; # These authors contributed eqully to this work

**Keywords:** Carbon tetrachloride, DNA, Fibrosis, Liver, Methylation

## Abstract

**Objective(s):**

Many persistent harmful stimuli can result in chronic liver diseases, which lead to about 2 million deaths per year in the whole world. Liver fibrosis was found to exist in all kinds of chronic liver diseases. Many studies suggested that DNA methylation was associated with the pathogenesis of liver fibrosis. This study aimed to quantitatively detect DNA methylation changes in the whole genome in fibrotic liver tissues of mice.

**Materials and Methods:**

Liver fibrosis was induced by intraperitoneal injection of carbon tetrachloride (CCl_4_) for 4 weeks. A genome-wide methylome analysis was performed using 850K BeadChips assays. The methylation status of 27 CpG dinucleotides located in 3 genes was detected by pyrosequencing to confirm chip data accuracy, and mRNA expressions of these 3 genes were examined by RT-qPCR methods.

**Results:**

A total of 130,068 differentially methylated sites (DMS, 58,474 hypermethylated, and 71,594 hypomethylated) between fibrotic liver tissues and control mice liver tissues were identified by the 850k BeadChips array. Consistency between pyrosequencing data and 850k BeadChips array data was observed (R=0.928; *P<*0.01). Apoptosis, positive regulation of transcription of Notch receptor target, and negative regulation of p38MAPK signal cascade activities were significantly enriched in the Gene Ontology (GO) analyses. Cholesterol metabolism, bile secretion, and more biosynthesis and metabolism pathways were enriched in KEGG pathway analyses. Ten key genes were identified by the Cytoscape plugin cytoHubba.

**Conclusion:**

7850 genes were found to have methylation change in fibrotic liver tissues of mice, which facilitates future research for clinical application.

## Introduction

Chronic liver diseases are a major health problem and result in about 2 million deaths per year all over the world (1). Many persistent stimuli can result in chronic liver diseases, such as hepatitis B and C virus, toxicity, dangerous metabolites, trauma, etc*.* (2). All chronic liver diseases were found to have progressive fibrogenesis in the liver. Liver fibrosis is characterized by the formation of a fibrous scar that originates from the accumulation of extracellular matrix (ECM) proteins, mostly collagens Type I, III, VI, V, and fibronectin (3). Fibrous scar has two functions: tissue protection and organ damage (4). It can protect against microbial invasion and help tissue repair (5). In minor or non-repetitive damage the deposition of ECM components only has a transient increase, facilitates the wound healing response, and then the scar can be dissolved by anti-fibrotic mechanisms (6). However, in chronic liver injury, it leads to distortion of the normal architecture of the liver, which inhibits regeneration and compromises the liver’s function. It eventually develops into liver cirrhosis and liver cancer (7).

 Liver fibrogenesis involves a multitude of molecular and cellular mechanisms. Cellular injury triggers oxidative stress and release of a series of inflammatory cytokines and growth factors. Signals of permanent inflammation and growth factors lead to the appearance of hepatic myofibroblast phenotype. Subsequently, the overproduction of extracellular matrix components results in the formation of fibrous scars (8, 9). Transforming Growth Factor Beta (TGF-β), WNT/β-catenin, the inflammasome (NLRP3)-Caspase1, and Platelet-Derived Growth Factor (PDGF) pathways were found to be key signaling pathways to promote fibrosis progression (10-12). Cell fate tracing techniques found that activated hepatic stellate cells are the predominant sources of myofibroblasts (13). Increasing evidence suggested that a small number of myofibroblasts can derive from bone marrow (14), portal fibroblasts (15), epithelial-to-mesenchymal transition (16), endothelial-to-mesenchymal transition (17) and mesothelial-to-mesenchymal transition (18). 

Epigenetics is the study of stable and inheritable alteration of gene expression without alterations in the DNA sequences. Epigenetics encompasses DNA methylation, histone modifications, non-coding RNAs, etc. (19). Many studies suggested that epigenetics is associated with the pathogenesis of fibrosis (20). DNA methylation is the most studied epigenetic modification (21). Increasing evidence has shown that aberrant DNA methylation plays an important role in the onset and progression of fibrosis (22). Hypermethylation of DNA in promoter regions can result in the transcriptional inactivation of genes, and hypomethylation of DNA is linked to increased gene expression. However, when DNA methylation is within the gene body and at CpG-poor sites, elevated expression has been observed (23). Methylation of DNA takes place on the 5th carbon of cytosine in a CpG dinucleotide. DNA methyltransferases (DNMTs) transfer a methyl group from S-adenosyl methionine to this carbon. DNMTs have two main categories: DNMT3A and DNMT3B catalyze de novo DNA methylation; DNMT1 maintains established methylation patterns during DNA replication. Depending on the DNA sequence context their functions overlap. Although DNA methylation is inheritable through generations, it can be erased by the ten-eleven translocation (TET) enzyme family. These enzymes initiate DNA demethylation either directly or through partners as an active process (21).

Fibrosis can occur in nearly all organs and is related to high morbidity. Organ fibrosis is responsible for up to 45% of all-cause mortality in the industrialized world (24). In many fibrotic diseases, there is no effective therapeutic schedule. There is a huge demand for anti-fibrotic treatment in clinics. DNA methylation patterns at specific CpG sites may be used as diagnosis and prognosis biomarkers in fibrotic disease (25). In order to comprehensively understand DNA methylation change and seek effective targets for future application, the 850K BeadChips were used to detect DNA methylation variation on a genome-wide scale in mice fibrotic liver tissues.

## Materials and Methods

***Generation of mice liver fibrosis***

C57BL/6 mice were purchased from Shanghai Laboratory Animals Inc. The mice were raised at the Experimental Animal Center of Henan Normal University. Feeding conditions were temperature (23±2 ^°^C), humidity (35±5%) with 12 hr day/night cycle, and free to get food and drinking water. 10-12 week-old male mice (n=9) were intraperitoneally injected CCl_4 _twice a week at 10 ul/g (CCl_4_/olive oil, 1:4) for 4 weeks to induce hepatic fibrosis. The same-age normal mice (n=9) were used as control. Twenty-four hours after the last injection, mice were anesthetized with 1% pentobarbital sodium (15 ml/kg) by abdominal cavity injection, and blood was released through the inferior vena cava, then liver tissues were harvested. Some of the livers were fixed with formalin and the other sections were preserved in a refrigerator at -80 ^°^C. All experimental procedures complied with animal ethics and the Animal Protection Law of China and were approved by the Academic Committee of Henan Normal University (Approval No.: HNSD-2020-02-05).

***Histological analysis***

Formalin-fixed liver tissue was embedded in paraffin and then cut into 5 cm sections. Hematoxylin and Eosin (H&E) staining and Masson’s trichrome (MT) staining were performed according to the manufacturer’s instructions. Images were captured for histological analysis under a light microscope (Nikon Eclipse TE2000-U, NIKON, Japan). 

***Genomic DNA and total RNA isolation***

Genomic DNA was extracted from the liver tissues using ONE-4-ALL Genomic DNA Mini-Preps Kit (BIO BASIC CANADA INC.) according to the technical manual. Total RNA was isolated using TRIZOL reagent following the specification (Sangon Biotech, China). Extracted DNA and RNA were evaluated by agarose gel electrophoresis at 1% concentration (GENVIEW); then the concentration was quantified using Nanodrop 2000 (Thermo). All samples were stored in the refrigerator at -80 ^°^C for future use.

***DNA methylation detection and data analysis***

500 ng DNA of each sample was used to bisulfite convert using EZ DNA Methylation Kits (Zymo Research, USA), and then converted products were put into the 850K BeadChips in accordance with the manufacturer’s guide and protocol (Illumina).

Raw sequencing data were first demultiplexed by bcl2fastq and then trimmed by fastp 0.19.4 (26) with default parameters in order to filter out bad reads, cut low-quality bases, trim all reads in front and tail, and cut adapters. The qualified reads were then mapped onto the reference genome (UCSC mm9) using the bisulfite sequence aligner Bismark 0.22.1 (27), which internally calls bowtie2 2.3.4.1 (28), and then PCR duplicates were removed by deduplicate_bismark.

The methylKit package (version 1.8.1) was used to identify Differential Methylation Sites (DMS) in R (version 3.5.2) (29). CpG with a minimum coverage ≥ 10 in all samples was considered in the following procedures. R methylKit calculateDiffMeth function was used to calculate differential methylation, in which by default logistic regression test was applied to evaluate the methylation difference between two groups, and then the SLIM method proposed by the methylKit package was used to correct the *P*-values for multiple testing. DMS was determined as methylation difference between groups ≥10% and Qvalue ≤0.05.

The edmr package was used to identify Differential Methylation Regions (DMR) in R, which is an optimized DMR analysis based on bimodal normal distribution model and cost function for regional methylation analysis (30). CpG with a minimum coverage ≥ 10 in all samples were considered, and each potential region at least includes 3 CpG sits and 1 differentially methylated CpG. DMR was determined as methylation difference between group ≥10% and Qvalue ≤0.05.

Enrichment analysis was conducted based on the Perl module using the Fisher test. Figures were plotted based on ggplot2 3.3.2.

***Confirmation of the differentially methylated sites***

 DNA was modified using the same kits mentioned above according to the manufacturer’s protocol. The bisulfite pyrosequencing primers were designed via PyroMark Assay Design Software 2.0 (Qiagen). The sequencing reaction and methylation level analysis were performed by The PyroMark Q96 ID System and software (Qiagen). The primer sequences for PCR and sequencing and reaction conditions were given in supplementary material 1.

***RT-qPCR***

The mRNA transcription of genes was detected by the RT-qPCR method. Briefly, RNA was reversely transcribed and qPCR analyses were immediately performed in the same test tube with one step RT-qPCR kit (Sangon Biotech, China) on an RT-qPCR system (LightCycler^R^480II, Roche, Switzerland). Each experiment has at least three repetitions. The thermal cycling settings were as follows: 50 ^°^C for 5 min, 95 ^°^C for 3 min, then 40 cycles of 95 ^°^C for 10 sec, and 60 ^°^C for 30 sec. Primer sequences were shown in Supplementary Table 1. The gene expressions were analyzed using the 2^−ΔΔCt^ method (31).

***Construction of the PPI network and screening of the key gene***

To demonstrate interactions among differential methylation genes (mean methylation difference between groups≥ 25%), a protein-protein interaction (PPI) network was constructed by the string database (32). Only combined scores≥0.4 between the interacting pairs were selected and displayed. The PPI network was visualized by the Cytoscape plugin cytoHubba adopting the ‘degree’ option. Then the MCC method in cytoHubba was used to identify 10 key genes.

## Results

***CCl***_4_ ***-induced mice liver fibrosis***

**Figure 1:**
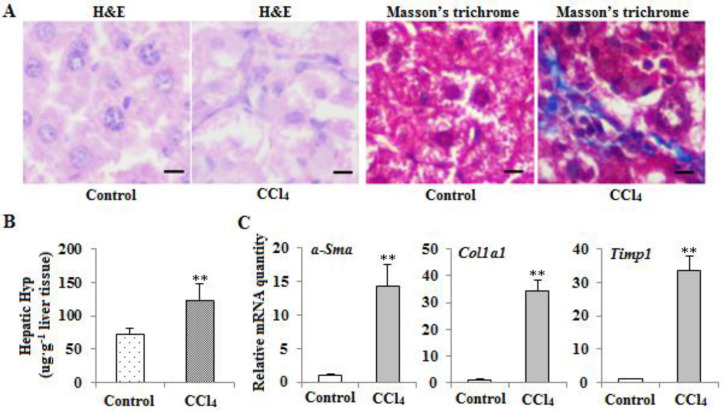
Confirmation of mice liver fibrosis induced by CCl_4_. (A) H&E and MT staining of liver sections (Scale bar, 50 μm; original magnification x200). (B) Content of hepatic HYP in both types of mice (**P<0.01, n=9). (C) The mRNA transcription of the fibrotic marker genes in liver tissues of both types of mice. The mRNA expression of a-Sma, Col1a1, and Timp1 was detected by RT-qPCR methods, and β-actin mRNA was used to normalize gene expression (**P<0.01, n=9)

To observe whether there is liver fibrosis after CCl_4_ treatment, we detected histopathological change in both groups by H&E and Masson’s trichrome staining. As shown in Figure 1A, there are obvious fibrosis formation and collagen deposition in the treatment group. Next, we examined the changes in hydroxyproline (HYP) content in liver tissues of the treatment group and control group mice. HYP content is higher in the CCl_4_ treatment group than in the control group (Figure 1B). Furthermore, the up-regulation of the classical fibrotic markers α-*Sma*, *Col1a1*, and *Timp1* was observed by RT-qPCR analysis (Figure 1C). These suggest that treatment of CCl_4_ has induced liver fibrosis.

***Global DNA methylation dynamics in the mice fibrotic liver tissues***

**Figure 2:**
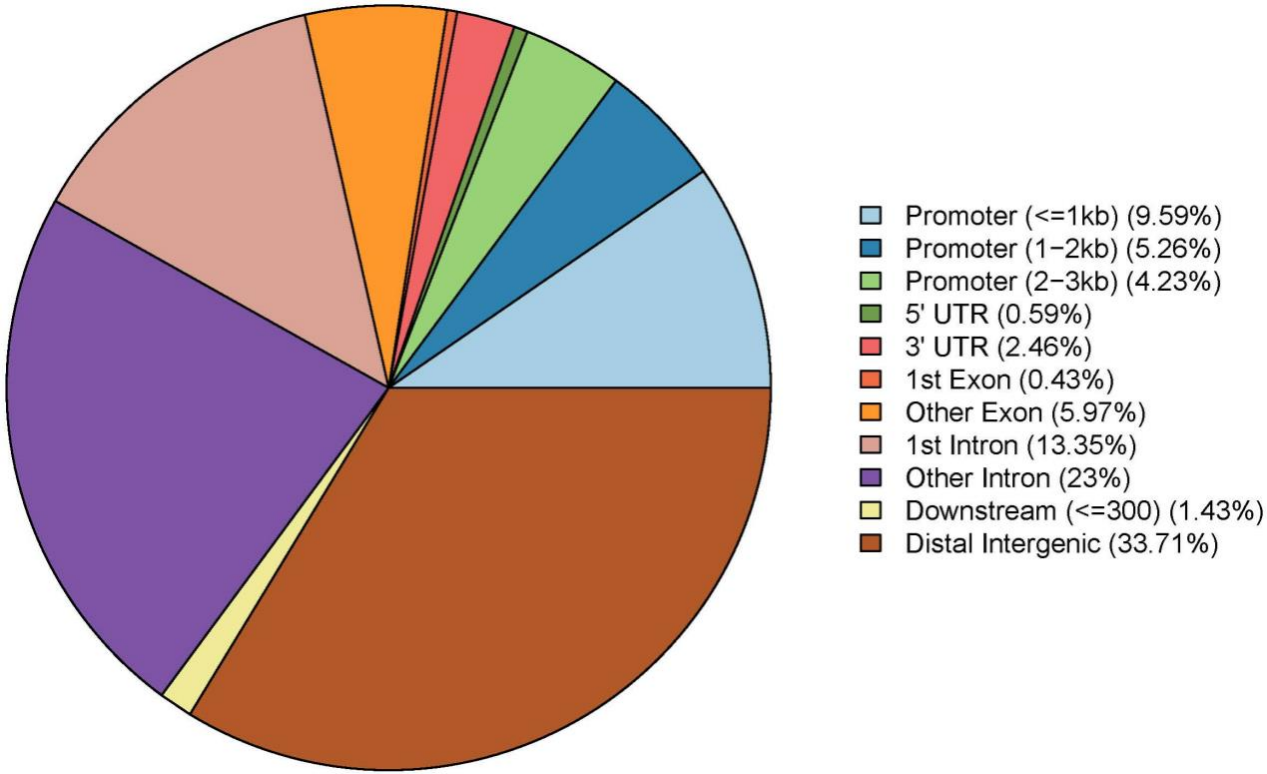
DMRs were located in different genomic regions

A total of 130,068 DMS (58,474 hypermethylated and 71,594 hypomethylated) that have a significant difference in methylation level between fibrotic liver tissues and control were identified with qvalue<0.05 and meth.diff>10. They were found to distribute in 25,550 DMR by ChIPseeker software analysis and involve 7850 genes. These DMRs were located in various positions of genes (Figure 2). As illustrated in Table 1, the DMS occurred in each chromosome. Almost all chromosomes have more hypomethylated DMS than hypermethylated DMS except that chromosome 18 is the opposite. In particular, 28 CpGs with the largest methylation difference between fibrotic liver tissues and control were identified (qvalue<0.05 and meth.diff>55), which were involved in 24 genes (Table 2).

***Technical verification of differential methylation loci by bisulfite pyrosequencing***

Twenty-seven of CpG sites with a significant difference in the promoter or exon of genes were selected for confirmation by bisulfite pyrosequencing using the same DNA. 11 CpG sites in exon of *Alpk1*, 6 CpG sites in promoter of *Cyp7a1 *and 1 CpG site in promoter of *Eng* were found to have striking difference (Table 3 *P*<0.05 or *P*<0.01). 9 CpG sites in promoter of Eng did not have a significant difference in statistics but they had the same change trend as the chip assays result (Supplementary Table 2). Pyrosequencing and DNA methylation change based on chip assays were significantly correlated with each other (Pearson’s correlation coefficient is 0.928 and *P*<0.01), suggesting that the chip data had been confirmed by pyrosequencing. Overall, many DNA methylation changes have been found in the DNA of fibrotic liver compared with the DNA of normal liver.

***Association of DNA methylation and gene mRNA transcription***

The mRNA changes of three genes confirmed to have methylation changes were detected by the RT-qPCR method. The correlation between the largest methylation difference site and gene expression was evaluated. Methylation change at 32502467 annotated to the promoter of *Eng* was found to have a negative correlation with its mRNA transcript (R= -0.769, *P*=0.016). Methylation change at 127398377 located at exon 4 of *Alpk1* was found to have a positive correlation with its expression (R=0.808, *P*=0.008). Methylation change at 6199856 located at the promoter of *Cyp7a1* does not correlate with its expression.

***Functional analysis of the differentially methylated genes***

**Figure 3:**
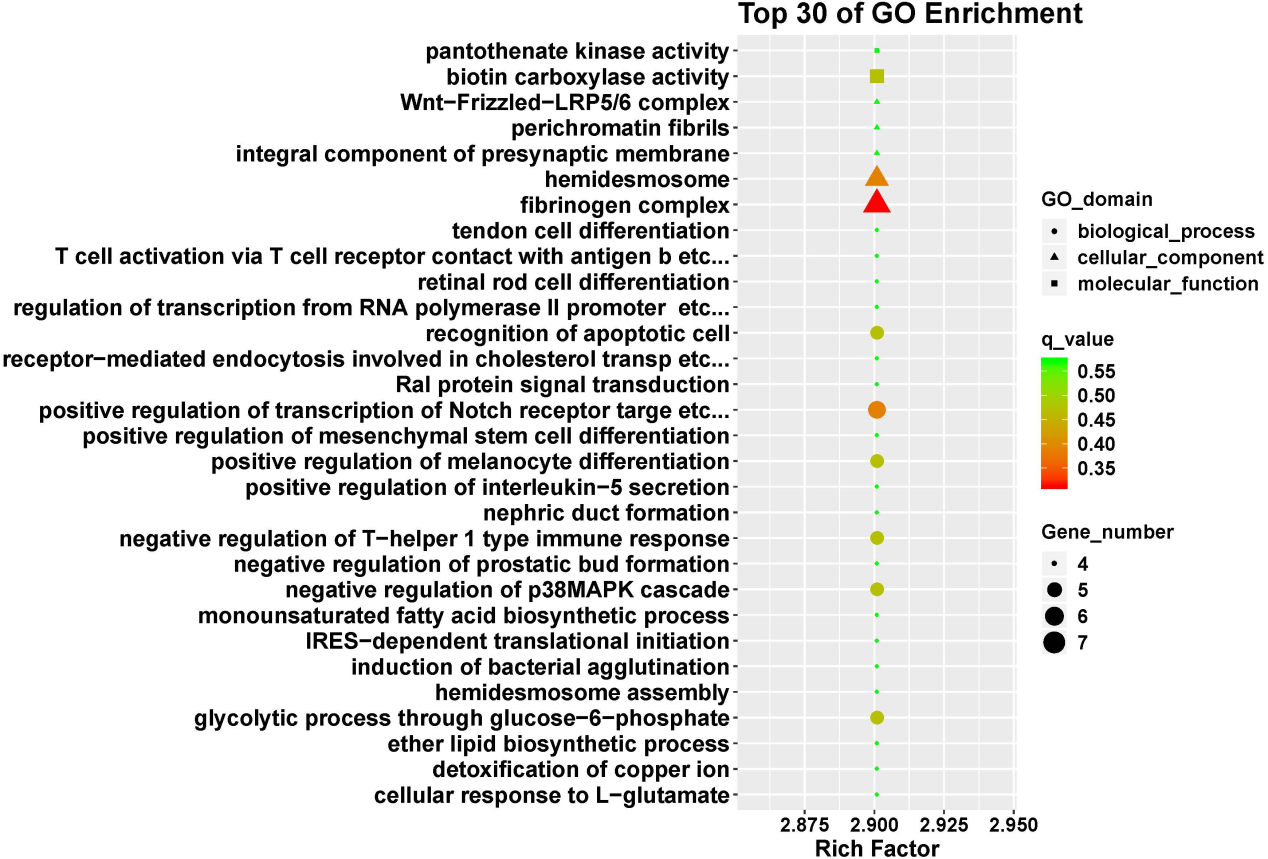
Top 30 significant GO terms in GO enrichment analysis of differentially methylated genes

**Figure 4:**
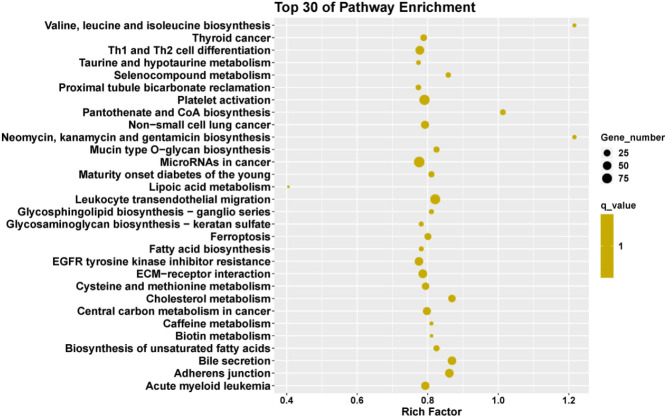
The top 30 pathway terms in the KEGG pathway enrichment analysis of differentially methylated genes

To explore possible functional enrichment of the DMR-related genes, GO and KEGG analyses were employed to classify these genes. Figures 3 and 4 present the top 30 of GO enrichment and KEGG enrichment, respectively; they can also be seen in supplementary Tables 3 and 4. The most enriched molecular functions were pantothenate kinase activity and biotin carboxylase activity. Cellular components include Wnt-Frizzled-LRP5/6 complex, perichromatin fibrils, fibrinogen complex components, etc. Regulation of transcription from RNA polymerase II promoter in response to oxidative stress, recognition of apoptotic cell, and positive regulation of transcription of Notch receptor target were enriched in biological process. Moreover, KEGG pathway analysis showed that valine, leucine, isoleucine biosynthesis, thyroid cancer, and taurine and hypotaurine metabolism-related pathways were involved in liver fibrogenesis.

***Construction of PPI network and identification of key genes***

**Figure 5:**
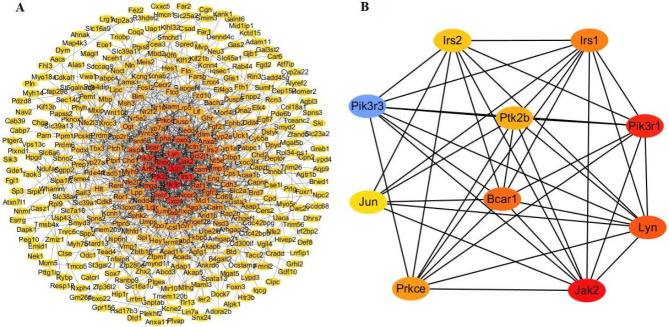
PPI network of differentially methylated genes. (A) PPI network of 384 genes. (B) Top 10 key differential methylation genes of the PPI network were identified by cytoHubba plugin. The node color changes according to the scores of the MCC algorithm

A PPI network was constructed using the string database. The network was further processed with Cytoscape 3.9.0 to visualize the interaction among genes more intuitively (Figure 5A). Then, the cytoHubba plugin in Cytoscape was used to identify the top 10 key genes using the MCC algorithm. The 10 key genes were *Irs1*, *Irs2*, *Pik3r3*, *Ptk2b*, *Pik3r1*, *Jun*, *Bcar1*, *Lyn*, *Prkce* and *Jak2* (Figure 5B).

DMRs: Differential methylation regions

**Table 1:** Differentially methylated sites (DMS) detected between fibrotic liver tissues and control

**Chr**	** HyperM DMS**	** HypoM DMS**	** Total DMS per Chr**	** % Whole genome** ^a^	**% HyperM DMS per Chr** ^b^	**% HypoM DMS per Chr** ^c^
**1**	3810	4677	8487	6.53%	44.89%	55.11%
**2**	3841	5114	8955	6.88%	42.89%	57.11%
**3**	2842	3642	6484	4.99%	43.83%	56.17%
**4**	3946	4529	8475	6.52%	46.56%	53.44%
**5**	3961	4878	8839	6.80%	44.81%	55.19%
**6**	3278	3594	6872	5.28%	47.70%	52.30%
**7**	2760	3827	6587	5.06%	41.90%	58.10%
**8**	3328	3592	6920	5.32%	48.09%	51.91%
**9**	2976	3576	6552	5.04%	45.42%	54.58%
**10**	3598	4228	7826	6.02%	45.97%	54.03%
**11**	4079	5995	10074	7.75%	40.49%	59.51%
**12**	2670	3129	5799	4.46%	46.04%	53.96%
**13**	2822	3177	5999	4.61%	47.04%	52.96%
**14**	2264	2720	4984	3.83%	45.43%	54.57%
**15**	2946	3390	6336	4.87%	46.50%	53.50%
**16**	1938	2525	4463	3.43%	43.42%	56.58%
**17**	2455	3618	6073	4.67%	40.42%	59.58%
**18**	2203	2102	4305	3.31%	51.17%	48.83%
**19**	2376	2492	4868	3.74%	48.81%	51.19%
**x**	369	771	1140	0.88%	32.37%	67.63%
**y**	4	7	11	0.01%	36.36%	63.64%
**ChrUn**	8	11	19	0.01%	42.11%	57.89%

^a^ Percentage of DMS per chromosome among the total DMS in genome-wide

^b^ Percentage of hypermethylated DMS relative to the total DMS per chromosome

^c ^Percentage of hypomethylated DMS relative to the total DMS per chromosome

HyperM: hypermethylated; HypoM: hypomethylated

**Table 2:** Top 28 differentially methylated sites (DMS) between fibrotic liver tissues and control (methylation differation>55)

**Hypermethylated No.**	**Position**	**UCSC_REFGENE_NAME**	**meth.diff**		** Hypomethylated No.**	**Position**	**UCSC_REFGENE_NAME**	**meth.diff **
1	90318822	***Spp2***	58.97		1	67710772	***Usp43***	-56.50
2	86440714	***Vmp1***	56.85		2	89217636	***Klhdc7b***	-65.45
3	115477416	***Mif4gd***	55.87		3	26651750	***Dusp1***	-67.65
4	66806333	***Ptk2b***	55.34		4	26651733	***Dusp1***	-66.08
5	34032027	***Kalrn***	57.60		5	12232473	***Agpat4***	-59.72
6	34032023	***Kalrn***	56.95		6	31344675	***Ubash3a***	-56.18
7	3659483	***Lrp5***	57.39		7	71096432	***Dlgap1***	-55.70
8	33086126	***Angptl2***	55.62		8	165230995	***Ocstamp***	-65.89
9	127398377	***Alpk1***	61.53		9	50999435	***Noct***	-59.52
10	27122509	***Nceh1***	58.70		10	95116629	***Cers2***	-58.51
11	11150267	***Ints8***	59.19		11	95116585	***Cers2***	-58.42
12	134406741	***Maco1***	56.10		12	95116618	***Cers2***	-55.23
13	71443598	***Mtmr10***	56.66		13	73263381	***Chsy1***	-56.67
14	63486769	***Nek1***	55.04		14	48515661	***Nnmt***	-58.53

**Table 3:** Pyrosequencing analysis was used to confirm DNA methylation levels detected on 850k BeadChip assays

**Chromosome**	**Position**	**Gene symbol**	**Gene region**	**p_value**
chr2	32502400	***Eng***	Promoter	0.1060
chr2	32502409	***Eng***	Promoter	0.4528
chr2	32502412	***Eng***	Promoter	0.9739
chr2	32502416	***Eng***	Promoter	0.0982
chr2	32502420	***Eng***	Promoter	0.6157
chr2	32502441	***Eng***	Promoter	0.0632
chr2	32502455	***Eng***	Promoter	0.0634
chr2	32502467	***Eng***	Promoter	0.0371
chr2	32502485	***Eng***	Promoter	0.2719
chr2	32502490	***Eng***	Promoter	0.1623
chr3	127398307	***Alpk1***	The 4th Exon	0.0001
chr3	127398327	***Alpk1***	The 4th Exon	0.0007
chr3	127398334	***Alpk1***	The 4th Exon	0.0001
chr3	127398349	***Alpk1***	The 4th Exon	0.0000
chr3	127398351	***Alpk1***	The 4th Exon	0.0025
chr3	127398361	***Alpk1***	The 4th Exon	0.0003
chr3	127398377	***Alpk1***	The 4th Exon	0.0000
chr3	127398380	***Alpk1***	The 4th Exon	0.0002
chr3	127398387	***Alpk1***	The 4th Exon	0.0021
chr3	127398393	***Alpk1***	The 4th Exon	0.0000
chr3	127398397	***Alpk1***	The 4th Exon	0.0087
chr4	6199802	***Cyp7a1***	Promoter	0.0059
chr4	6199813	***Cyp7a1***	Promoter	0.0014
chr4	6199830	***Cyp7a1***	Promoter	0.0005
chr4	6199856	***Cyp7a1***	Promoter	0.0001
chr4	6199870	***Cyp7a1***	Promoter	0.0022
chr4	6199892	***Cyp7a1***	Promoter	0.0071

## Discussion

DNA methylation is an important means to regulate gene transcription and development. Aberrant DNA methylation can lead to inappropriate gene expression and implicate in a variety of human disease processes including fibrosis and cancer (33). DNA methylation changes have been reported to be associated with the progression of liver fibrosis (22). Table 1 demonstrated that the DNA methylation declined as a whole; there are more hypomethylated DNA than hypermethylated DNA in mice fibrotic liver tissues. DNA hypomethylation was thought to lead to genomic instability (34). Our result is consistent with global DNA methylation change in early-stage liver fibrosis (35). Although both hypomethylation and hypermethylation occurred in the DNA of mice fibrotic liver tissues, previous reports indicate inhibition of DNA methylation can attenuate the progression of renal (36) and liver fibrosis (37), and hepatic fibrogenesis is related to hypermethylation of DNA (38). Perhaps hypermethylation plays a more important role in the development of liver fibrosis.

We confirmed 18 CpG dinucleotides distributed in 3 genes (*Eng, Alpk1*, and *Cyp7a1*) by pyrosequencing. Site 32502467 was located at the promoter of *Eng* (Endoglin). Its methylation decreased in liver fibrosis, which has a negative correlation with *Eng* mRNA transcript. It agrees that hypermethylation of DNA in promoters can lead to the silence of genes (23). ENDOGLIN is a transmembrane protein, which acts as an ancillary receptor for several members of the TGF-β superfamily and BMP superfamily of cytokines. ENDOGLIN functions by modulating TGF-β and BMP signaling, two pathways that are proven to have an important effect on inflammation, tissue repair, and fibrogenesis (39, 40). A lot of evidence shows that ENDOGLIN is related to angiogenesis. ENDOGLIN expression was observed to be increased in the endothelium in the active angiogenesis process (41). A vascular malformations disease, hereditary hemorrhagic telangiectasia type-1 (HHT-1) is related to deficiency in *Endoglin* expression. The lack of *Endoglin* results in impaired neoangiogenesis and vascular malformations because of the alterations of endothelial cell physiology (39). Site 127398377 was located at exon 4 of *Alpk1*. Its methylation increased in liver fibrosis, which has a positive correlation with *Alpk1* mRNA expression. It is consistent with a previous report that DNA methylation within the gene body was related to elevated expression (23). ALPK1 is a ɑ-protein kinase, which is a member of a family of atypical protein kinases (42). It was found to be a susceptibility marker gene to gout, chronic kidney disease, myocardial infarction, and type 2 diabetes mellitus. It can also regulate inflammation (43). A recurrent heterozygous variant in *Alpk1* can cause autosomal dominant retinal dystrophy syndrome (44). *Alpk1*-mutant was associated with motor coordination deficits (45). Site 6199856 was annotated to the promoter of *Cyp7a1*. Its methylation increased in liver fibrosis, which has no association with *Cyp7a1* transcription. The expression of a gene was influenced by multiple mechanisms such as DNA mutations, miRNA, and lincRNA; perhaps other pathways have more important effects on the expression of *Cyp7a1*. CYP7A1 is a liver-specific enzyme that catalyzes the rate-limiting step in the bile acid biosynthetic pathway. Deficiency of *Cyp7a1* results in a strikingly decreased output of BAs, and it was related to hypercholesterolemia and premature atherosclerosis (46). Overexpression of *Cyp7a1* in transgenic mice liver reduced cholesterol content in the liver and plasma and prevents atherosclerosis (47); transgenic mice were resistant to high-fat diet-induced fatty liver, obesity, and insulin resistance, and kept triglyceride, cholesterol, and bile acid homeostasis (48). 

Meanwhile, we found many genes detected with methylation changes in 850K BeadChips were confirmed by other experiments. Yang and colleague reported that DNA hypermethylation of *Ptch1* was involved in decreased *Ptch1* expression during rats’ hepatic fibrosis (49). Yoshida and colleagues demonstrated the severity of liver fibrosis has strong relevance with *SOCS1* gene methylation in 200 patients with chronic liver disease (50). The *Spp1* enhancer was found to be hypo-methylated and its expression increased in early-stage liver fibrosis (35). *Fgfr3*, *Camk4*, *Gpx4*, *Hoxd3*, and *Prkcb* were verified to be hyper-methylated in hepatic fibrosis of mice induced by CCl_4_ for 8 weeks (51). In addition, some genes were also found to have methylation changes in liver cancer. Among them 419 genes were found in the TCGA database (52); 34 genes were thought to be oncogenes in the ONGene database (53) (supplementary material 2). Perhaps they are a kind of precancerous change and are useful for predicting future risk. 

The top 10 key genes found by the cytoHubba plugin all have methylation changes in liver cancer samples in the TCGA database. Among them 6 genes (*Irs2*, *Ptk2b*, *Pik3r1*, *Jun*, *Prkce*, and *Jak2*) were thought to be oncogenes in the ONGene database; there is 190 cancer-related literature references for *Jun* and 15 for *Jak2*. *SEPT9* methylated DNA test has been approved by FDA for colorectal cancer screening (54). Perhaps *Jun* and *Jak2* changed methylations are potential biomarkers for predicting liver cancer. Among these 10 key genes *Pik3r3*, *Pik3r1,* and *Jun *participated in TGF-β signaling, *Jun* participated in BMP signaling, *Jun* and *Prkce* participated in WNTS signaling, *Pik3r3*, *Pik3r1*, *Jun*, and *Jak2* participated in PDGF signaling. Perhaps they play the most important role in liver fibrogenesis.

To investigate the function of differential methylation genes in liver fibrosis, GO enrichment analysis was carried out. The result indicated that the biological processes were closely associated with detoxification of copper ion, recognition of apoptotic cells, positive regulation of transcription of Notch receptor target, and negative regulation of p38MAPK cascade activities. Detoxification was an important function of the liver (55). Apoptosis (56), Notch (57), and p38MAPK (58) pathways are important profibrotic signal pathways. Fibrinogen complex as a cellular component probably contributes to fibrosis formation. Meanwhile, KEGG analysis showed that enrichment of differential methylation genes mainly focuses on ECM-receptor interaction, cholesterol metabolism, and bile secretion pathways. There are more biosynthesis and metabolism pathways. DNA methylation may, by affecting cholesterol metabolism and bile secretion, influence liver function. ECM-receptor interaction may be related to fibrotic scar formation.

## Conclusion

This study provides new evidence that DNA methylation is involved in the progression of liver fibrosis. The number of hypomethylation is larger than that of hypermethylation, but hypermethylation probably plays a more important role in liver fibrogenesis. Methylation change of many genes in liver fibrosis has also been found in liver cancer; some of them may be useful for predicting carcinogenesis risk. It facilitates future research to find more accurate targets for clinical diagnosis and treatment of liver fibrosis.

## Authors’ Contributions

DML conceived, designed, and conducted the experiments. XSG, WYZ, JYC, and CX conducted the experiments. DML and XSG analyzed data and wrote the manuscript. GYY administrated and supervised the project, and reviewed and edited the manuscript. All authors read and approved the final version of the manuscript.

## Data availability

Methyl-seq data reported in this paper are available in the Gene Expression Omnibus database (GEO, https://www.ncbi.nlm.nih.gov/geo/, Accession number: GSE 193182).

## Conflicts of Interest

The authors declare that they have no conflicts of interest.
